# Meta-analysis of nanoparticle albumin-bound paclitaxel used as neoadjuvant chemotherapy for operable breast cancer based on individual patient data (JBCRG-S01 study)

**DOI:** 10.1007/s12282-021-01238-9

**Published:** 2021-04-03

**Authors:** Manabu Futamura, Mari Oba, Norikazu Masuda, Hiroko Bando, Morihito Okada, Yutaka Yamamoto, Takanori Kin, Toshiaki Saeki, Takeshi Nagashima, Takashi Kuwayama, Uhi Toh, Akira Hirano, Masafumi Inokuchi, Kazuhiko Yamagami, Yutaka Mizuno, Yasuyuki Kojima, Takahiro Nakayama, Hiroyuki Yasojima, Shinji Ohno

**Affiliations:** 1 Department of Surgical Oncology, Gifu University Graduate School of Medicine, Gifu, 501-1194 Japan; 2 Department of Medical Statistics, Toho University, Tokyo, 143-8540 Japan; 3 Department of Surgery, Breast Oncology, National Hospital Organization Osaka National Hospital, Osaka, 540-0006 Japan; 4 Department of Breast-Thyroid-Endocrine Surgery, Tsukuba University, Tsukuba, 305-8576 Japan; 5 Research Institute for Radiation Biology and Medicine, Hiroshima University, Hiroshima, 734-8553 Japan; 6 Department of Breast and Endocrine Surgery, Graduate School of Medical Sciences, Kumamoto University, Kumamoto, 860-8556 Japan; 7 Department of Breast Surgery, Hiroshima City Hiroshima Citizens Hospital, Hiroshima, 730-8518 Japan; 8 Department of Breast Oncology, Saitama Medical University International Medical Center, Saitama, 350-1298 Japan; 9 Department of General Surgery, Graduate School of Medicine, Chiba University, Chiba, 260-8677 Japan; 10 Division of Breast Surgical Oncology, Showa University, Tokyo, 142-8666 Japan; 11 Department of Surgery, Kurume University School of Medicine, Kurume, 830-0011 Japan; 12 Department of Breast Surgery, Medical Center East, Tokyo Women’s Medical University, Tokyo, 116-8567 Japan; 13 Department of Breast and Endocrine Surgery, Kanazawa Medical University, Ishikawa, 920-0293 Japan; 14 Department of Breast Surgery and Oncology, Shinko Hospital, Kobe, 651-0072 Japan; 15 Department of Breast Surgery, Yokkaichi Municipal Hospital, Yokkaichi, 510-8567 Japan; 16 Division of Breast and Endocrine Surgery, Department of Surgery, St. Marianna University School of Medicine, Kawasaki, 216-8511 Japan; 17 Department of Breast and Endocrine Surgery, Osaka International Cancer Institute, Osaka, 541-8567 Japan; 18 Breast Oncology Center, Cancer Institute Hospital, Tokyo, 135-0063 Japan

**Keywords:** Nanoparticle albumin-bound paclitaxel, Meta-analysis, Individual patient data, Pathological complete response, HER2-rich

## Abstract

**Background:**

Nanoparticle albumin-bound paclitaxel (nab-PTX), a novel taxane formulation, was developed to avoid cremophor/ethanol-associated toxicities including peripheral neuropathy and hypersensitivity. At least 35 phase II studies using combined nab-PTX and anthracycline in neoadjuvant settings are registered in Japan. We analyzed the efficacy and safety of nab-PTX based on patient characteristics in these studies.

**Methods:**

We conducted a meta-analysis using individual patient data (IPD) to investigate the average efficacy of nab-PTX-containing regimens as neoadjuvant chemotherapy for operable breast cancer. IPD were provided by principal investigators who agreed to participate. The primary endpoint was pathological complete response (pCR) rate of each breast cancer subtype.

**Results:**

We analyzed the data of 16 studies involving 753 patients. The overall crude frequencies of pCR (ypT0 ypN0, ypT0/is ypN0, and ypT0/is ypNX) were 18.1, 26.0, and 28.6%, respectively. Specifically, the frequencies were 6.7, 10.2, and 13.4% for luminal (*n* = 343); 40.5, 63.5, and 68.9% for human epidermal growth factor receptor 2 (HER2)-rich, (*n* = 74); 21.9, 40.6, and 42.7% for luminal/HER2 (*n* = 96); and 26.3, 31.5, and 32.3% for triple-negative breast cancers (TNBC) (*n* = 232). The multivariate analyses indicated that HER2 positivity, TNBC, high Ki-67, high nuclear grade, and weekly nab-PTX administration were significantly associated with the pCR. The proportion of hematological toxicities (neutropenia (39.7%) and leukopenia (22.5%)), peripheral sensory neuropathy (9.7%), myalgia (5.7%), and arthralgia (4.7%) was higher than grade 3 adverse events, but most patients recovered.

**Conclusions:**

Nab-PTX is a safe and acceptable chemotherapeutic agent in neoadjuvant settings, particularly for aggressive cancers. UMIN-CTR#: UMIN000028774

**Supplementary Information:**

The online version contains supplementary material available at 10.1007/s12282-021-01238-9.

## Introduction

Although taxane is a current gold standard chemotherapeutic agent for breast cancer (BC), adverse events (AEs) such as peripheral neuropathy and hypersensitivity are often problematic for patients. Nanoparticle albumin-bound paclitaxel (Nab-PTX) is a novel taxane formulation that was developed to avoid the toxicities associated with cremophor/ethanol co-solvents, such as the aforementioned peripheral neuropathy and hypersensitivity reactions [1, 2]. Nab-PTX showed higher tumor suppression in a mouse model than conventional paclitaxel used at a high concentration [3]. As taxol plays an important role in BC therapy, nab-PTX was first utilized for metastatic BC, resulting in longer progression-free survival (PFS) than that achieved with either paclitaxel or docetaxel [4, 5]. Nab-PTX has been reported to induce specific AEs such as arthralgia, myalgia, and peripheral neuropathy, but they are transient and controllable [5]. These reports suggest wide applications of nab-PTX for BC therapy. However, a few large-scale phase III studies using a nab-PTX-containing regimen in a neoadjuvant setting have reported remarkable findings that nab-PTX is more effective than paclitaxel [6].

In Japan, nab-PTX was approved for use and insurance coverage in July 2010; it has been widely used in both neoadjuvant and metastatic settings. At least 35 phase II neoadjuvant studies using nab-PTX have been registered in the University Hospital Medical Information Network-Clinical Trial Registry (UMIN-CTR) [7]. Of these, several studies using a combination of nab-PTX and anthracycline as neoadjuvant chemotherapy (NAC) have been reported [8–13]. However, these studies have not progressed to phase III randomized controlled trials (RCTs) and their sample sizes have been small. In addition, the results varied because the distribution of patient characteristics differed among the studies. Thus, to estimate the efficacy and safety of nab-PTX precisely in a neoadjuvant setting, we aimed to extract individual patient data (IPD) from studies on nab-PTX-containing regimens registered in UMIN-CTR and analyze the efficacy and safety of nab-PTX based on patient characteristics in a meta-analysis [14].

## Materials and methods

### Specific criteria

This study is a collaborative meta-analysis of phase II trials using IPD to summarize published and unpublished evidence on the efficacy of nab-PTX-containing regimens. Patients with operable BC (cStages I–III) who received NAC with nab-PTX were included. The primary endpoint was the pathological complete response (pCR) rate in each subtype. Clinical subtypes were defined by immunohistochemical evaluation according to the General Rules for Clinical and Pathological Recording of Breast Cancer (17th edition) based on the UICC-TNM classification [15]. The three definitions of pCR were as follows: (1) ypT0 ypN0, no invasive or noninvasive residual in the breast and lymph nodes; (2) ypT0/is ypN0, no invasive residual in the breast and lymph nodes; and (3) ypT0/is ypNX, no invasive residual in the breast [16]. The secondary endpoints were frequency of greater than Grade 3 AEs (≥ G3), total dose of nab-PTX (mg/body), disease-free survival (DFS), and overall survival (OS). DFS was defined as the time to relapse or all-cause death from the date of trial registration. OS was defined as the time to all-cause death from the date of registration. This study is registered at UMIN-CTR under UMIN000028774.

### Search strategy and eligibility criteria

The inclusion criteria were as follows: (1) phase II clinical trial(s) started after July 2010, (2) principal investigators (PIs) agreed to provide IPD, (3) Nab-PTX-containing regimens were used for NAC in chemo-naïve, operable BC patients, (4) registered at UMIN-CTR, (5) approved by an ethics committee, (6) more than 10 patients were enrolled, and (7) clinical study had already been completed (unpublished data were available). Our inclusion criteria for safety analysis using IPD were (1) operable (Stages I–III), (2) patients with no previous treatment, and (3) patients who underwent at least one cycle of each regimen. Our inclusion criteria for efficacy analysis were (1) patients who underwent surgery and (2) progressive disease (PD).

### Data collection

Data collection from the clinical trials was approved by each ethical committee and consent was obtained from the sponsor if necessary. All the studies provided patients with an opportunity to opt-out before data submission. Data pertaining to the following variables were requested from all studies: age, menopause, histology of pre/post NAC [estrogen receptor (ER), progesterone receptor (PgR), HER2, Ki-67, nuclear grade (NG), and histological grade (HG)], regimen, doses of NAC agents, surgical methods, image evaluation, AEs ≥ G3, effect of NAC, DFS, and OS.

### Assessment of studies

Before analysis, we checked the risk of bias using the Risk of Bias Assessment Tool for Nonrandomized Studies (RoBANS) and Cochrane training [17, 18]. Next, we constructed a forest plot and evaluated the heterogeneity of the pCR rates among the studies. The *I*^*2*^ statistic, which is the ratio of heterogeneity to total variance in the pCR rates among all studies, was calculated. A funnel plot was constructed to assess publication bias, which displayed the relationship between the study size and effect size.

### Statistical analysis using IPD

The pCR rate and 95% confidence interval (CI) were calculated for each study, for all patients, and for subgroups. The preplanned subgroup variables were menopause, age, NG, Ki-67, clinical stage, use of nab-PTX, clinical response, subtype, and HER2 status. Forest plots were used to display the pCR rates by subgroups. The association of clinical variables with the achievement of pCR was assessed using univariate and multivariable logistic models and expressed as odds ratios (ORs). A multiple imputation approach was used to manage missing clinical variables in the multivariate logistic model. The proportion of AE ≥ G3 and average total dose of nab-PTX per patient were also calculated. The difference in the proportion of AEs between q3w (every 3 weeks) and weekly nab-PTX was tested using the Chi-square test. The total dose of nab-PTX administered per patient was compared between nab-PTX regimens using *t* tests. DFS and OS were summarized using the Kaplan–Meier method and compared using the log-rank test. Hazard ratios (HRs) were calculated using a crude Cox proportional hazards model. All statistical tests were two-sided, and results with *p* < 0.05 were considered statistically significant. Statistical multiplicity was not adjusted. All statistical analyses were performed using SAS software version 9.4 (SAS Institute, Cary, NC) and R package “metaphor.”

## Results

### Characteristics of clinical trials

**Fig. 1:**
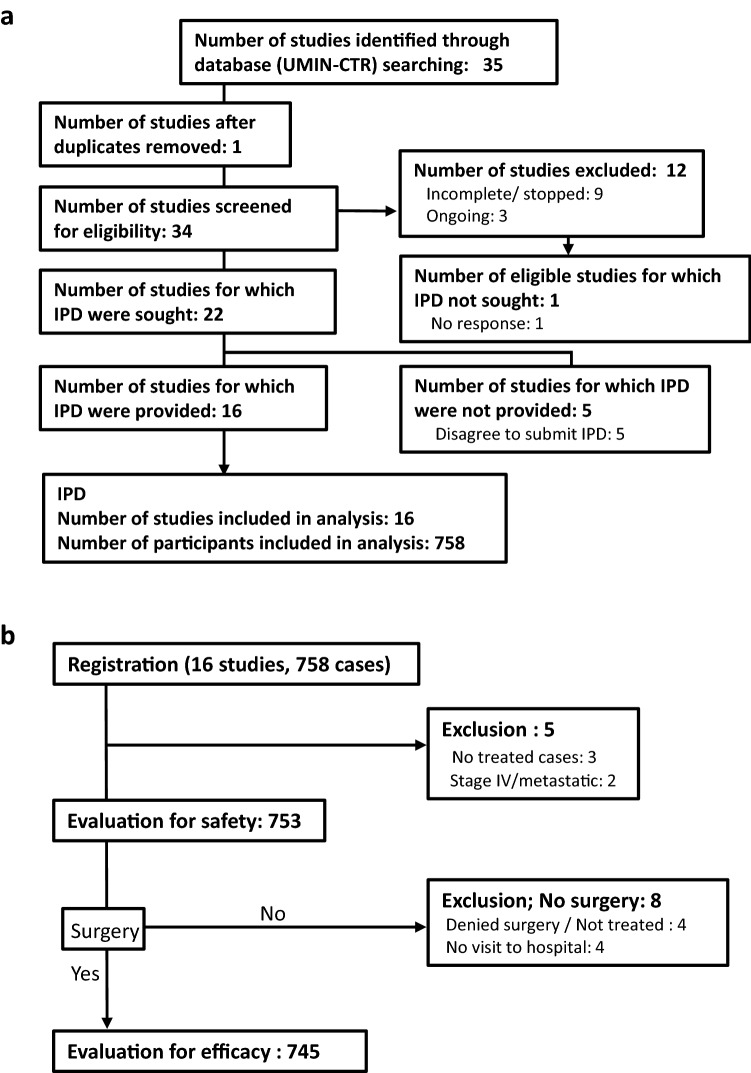
PRISMA flow diagrams for the meta-analysis. **a** Identification of studies. **b** Inclusion/exclusion of patients

We found 35 studies in the UMIN-CTR, which were reviewed using the PRISMA IPD flow diagram shown in Fig. 1a [19]. Twelve studies were either incomplete or ongoing, and one was a duplicate; six studies failed to provide IPD. Thus, the datasets of 16 studies (6 published [8–13] and 10 unpublished) involving 758 patients were selected for further analysis. All included studies were phase II; 15 were single-arm and one was an RCT. The protocol regimens utilized both nab-PTX and anthracycline. Nab-PTX was administered either q3w (11 studies) or weekly (5 studies). Thirteen studies administered nab-PTX followed by anthracycline, and three studies administered anthracycline followed by nab-PTX (Table 1). For safety evaluation, 753 patients were analyzed because three patients did not receive treatment, and two metastatic cases were excluded. For efficacy evaluation, 745 patients were analyzed because four patients who denied surgery or treatment and four patients who did not visit the hospital were excluded (Fig. 1b).

**Table 1:** Study list in this meta-analysis

No.	Study group	UMIN ID	Study name	Case	Protocol	Dose of nab-PTX (mg/m^2^)	Reference	All data provided (*N* = 758)															
								Age mean (SD)	Distribution of subtypes (%)				Nuclear grade (%)				Pre Ki-67 mean (SD)	PS 0 (%)	cStage (%)			cCR (%)*	pCR (%)*
									Luminal	HER2-rich	Luminal-HER2	Triple negative	1	2	3	Unknown			I	IIA, B	IIIA–C	cCR	ypT0/is ypN0
1	Gifu University	UMIN000028774	PerSeUS BC01	54	HER2(−): nab-PTX(q3w)*4 → EC (q3w)*4 HER2( +): nab-PTX + TZ(q3w)*4 → EC (q3w)*4	260 mg/m^2^ q3w	[8]	52.7 (9.7)	35%	6%	35%	24%	6%	19%	61%	15%	52.4 (18.1)	100%	9%	85%	6%	17%	22%
2	Kurume University	UMIN000010504	KSCOG-BC-07	33	FEC (q3w)*4 → nab-PTX(q3w)*4 FEC(q3w)*4 → nab-PTX + TZ(weekly)*12	260 mg/m2 q3w 100 mg/m2 weekly		54 (9.9)	48%	9%	30%	12%	30%	24%	27%	18%	40.5 (18.7)	100%	6%	42%	52%	34%	24%
3	Kanazawa University	UMIN000010579		51	nab-PTX + TZ(q3w)*4 → FEC (q3w) *4	260 mg/m^2^ q3w		53.8 (10.6)	0%	49%	51%	0%	4%	6%	22%	69%	33.3 (21.4)	92%	10%	55%	35%	27%	43%
4	Kyusyu Breast Cancer Study Group	UMIN000030692	KBC-SG 1103	39	HER2(−): nab-PTX(q3w)*4 → FEC (q3w)*4 HER2( +): nab-PTX + TZ(q3w)*4 → FEC (q3w)*4	260 mg/m^2^ q3w		51.6 (9.8)	41%	13%	13%	33%	15%	18%	54%	13%	46.4 (25.9)	100%	5%	62%	33%	21%	37%
5	Hiroshima City Hiroshima Citizens Hospital	UMIN000009733		41	HER2(−): nab-PTX(q3w)*4 → FEC (q3w)*4 HER2( +): nab-PTX + TZ(q3w)*4 → FEC (q3w)*4	260 mg/m^2^ q3w		53.7 (11.6)	41%	5%	17%	37%	20%	27%	54%	0%	33 (18.4)	100%	0%	68%	32%	10%	20%
6	Hiroshima University	UMIN000007180	TRI-ABC-FEC trial	55	HER2(−): nab-PTX + CPA(q3w)*4 → FEC (q3w)*4 HER2( +): nab-PTX + CPA + TZ(q3w)*4 → FEC (q3w)*4	260 mg/m^2^ q3w	[9]	49.5 (10.7)	45%	13%	20%	22%	7%	27%	65%	0%	68.2 (26.5)	100%	15%	56%	29%	44%	39%
7	Saitama Medical University	UMIN000013513		53	nab-PTX(q3w)*4 → EC (q3w) *4	260 mg/m2 q3w	[10]	55.5 (11.1)	64%	0%	0%	36%	68%	23%	6%	4%	24.1 (14.9)	100%	0%	51%	49%	4%	4%
8	Yokkaichi Municipal Hospital	UMIN000032153		46	HER2(−): nab-PTX(q3w)*4 → EC (q3w)*4 HER2( +): nab-PTX + TZ(q3w)*4 → EC (q3w)*4	260 mg/m^2^ q3w		52.8 (11.5)	2%	13%	26%	59%	28%	20%	39%	13%	49.6 (23.8)	100%	22%	70%	9%	56%	44%
9	Showa University	UMIN000005388		51	nab-PTX(weekly)*12 → FEC (q3w)*4	100 mg/m^2^ weekly	[11]	49.6 (9)	67%	0%	0%	33%	47%	16%	33%	4%	30.9 (24.2)	100%	0%	98%	2%	8%	20%
10	Shinko Hospital	UMIN000020994		23	nab-PTX(q3w)*4 → FEC (q3w) *4	260 mg/m^2^ q3w		60.2 (10.9)	13%	43%	0%	43%	22%	13%	22%	43%	43.7 (21.5)	100%	4%	91%	4%	57%	48%
11	St. Marianna University	UMIN000005704		37	nab-PTX(weekly)*12 → FEC (q3w)*4	150 mg/m^2^ weekly	[12]	50.1 (9.4)	62%	0%	0%	38%	0%	0%	0%	100%	43.3 (22.5)	97%	0%	68%	32%	14%	24%
12	Chiba University	UMIN000007724	PINK-BC study	16	FEC (q3w)*4 → nab-PTX(q3w)*4	260 mg/m^2^ q3w	[13]	52.2 (11)	50%	0%	0%	50%	0%	0%	0%	100%	55.9 (28.9)	100%	0%	38%	63%	13%	13%
13	Kinki Breast Cancer Study Group	UMIN000008085	KBCRG-TR 1213	64	nab-PTX(q3w)*4 → FEC (q3w) *4	260 mg/m^2^ q3w		49.3 (9.4)	63%	0%	0%	38%	17%	20%	63%	0%	46.1 (24.7)	100%	6%	77%	17%	32%	17%
14	Kinki Breast Cancer Study Group	UMIN000012909	KBCRG-TR 1215	125	nab-PTX(q3w)*4 → FEC (q3w) *4	260 mg/m^2^ q3w		50.7 (9)	64%	0%	0%	36%	12%	19%	54%	15%	47.4 (24.7)	100%	6%	88%	6%	21%	17%
15	Tsukuba University	UMIN000006053		30	HER2(−): nab-PTX(weekly)*9 → FEC (q3w)*4 HER2( +): nab-PTX + TZ(weekly)*12 → FEC (q3w)*4	125 mg/m^2^ weekly		55 (9.9)	53%	13%	3%	30%	20%	30%	50%	0%	46.2 (26.9)	90%	7%	73%	20%	21%	38%
16	Tokyo Women’s Medical University Medical Center East	UMIN000007648		40	HER2(−): EC(q3w)*4 → nab-PTX(weekly)*12	125 mg/m^2^ weekly		53.8 (10.7)	48%	25%	15%	13%	10%	15%	33%	43%	34.8 (28.6)	100%	3%	70%	28%	28%	33%
		Total		758				52.2 (10.3)	46%	10%	13%	31%	19%	18%	41%	22%	44.3 (25.5)	99%	6%	71%	22%	25%	26%

*SD* standard deviation. All data (*N* = 758) have been registered. *N = 745 for clinical complete response (cCR) and pCR evaluation

Effectively analyzed populations in sixteen studies are shown

We were provided all study protocols and IPD data (with a few missing data points) by each principal investigator; the data were subjected to a quality check using RoBANS (Suppl. Fig. 1). Eventually, the risk of bias for the meta-analysis of non-RCTs was deemed to be moderate. The I^2^ statistic (68.8%) indicated a moderate heterogeneity among the 16 studies (Suppl. Fig. 2a). The funnel plot showed that most of the studies were distributed symmetrically, except for two small studies that reported low pCR rates (Suppl. Fig. 2b). Table 1 shows the characteristics of the clinical trials. The two studies that reported the low pCR rates included only luminal or triple-negative subtypes, and higher proportions of cStage III than the other studies. The distribution of patient characteristics varied among the trials.

### Characteristics of the patients

The characteristics of the patients are summarized in Table 2. All HER2-positive cases, except four (including luminal/HER2 cases), were treated by the combination of nab-PTX and trastuzumab. Of the 758 patients (mean age 52.2 years), the number of patients with cStages I, IIA, IIB, IIIA, IIIB, and IIIC was 47 (6.2%), 260 (34.5%), 279 (37.1%), 88 (11.7%), 35 (4.6%), and 44 (5.8%), respectively. Luminal, HER2-rich, luminal/HER2, and TNBC subtypes were observed in 347 (46.1%), 75 (10.0%), 96 (12.7%), and 235 (31.2%) patients, respectively. Ki-67, NG, and HG were not routinely evaluated in some clinical studies. Most HER2-rich populations involved high-NG, high Ki-67 (≥ 40%; median value was 40%), and the use of trastuzumab. The patient characteristics after NAC are shown in Suppl. [20, 21].

**Table 2:** Patient characteristics for evaluation of the total population and each subtype

		Cases (%)	Luminal (%)	HER2-rich (%)	Luminal/HER2 (%)	Triple negative (%)
Sex	Female	753 (100.0%)	347 (100%)	75 (100%)	96 (100%)	235 (100%)
Age	mean (SD)	52.2 (10.3)	51.5 (9.9)	54.9 (10.0)	51.6 (10.6)	52.7 (10.7)
	< 40	102 (13.6%)	47 (13.5%)	8 (10.8%)	15 (15.6%)	32 (13.6%)
	40–59	439 (58.4%)	211 (60.8%)	43 (58.1%)	55 (57.3%)	130 (55.3%)
	60 ≤	211 (28.1%)	89 (25.6%)	23 (31.1%)	26 (27.1%)	73 (31.1%)
	UK	1	0	1	0	0
Menstruation	Premenopausal	362 (48.2%)	188 (54.2%)	24 (32.4%)	41 (43.2%)	109 (46.4%)
	Postmenopausal	389 (51.8%)	159 (45.8%)	50 (67.6%)	54 (56.8%)	126 (53.6%)
	UK	2	0	1	1	0
Performance status	0	745 (99.2%)	345 (99.4%)	73 (98.6%)	94 (98.9%)	233 (99.1%)
	1	6 (0.8%)	2 (0.6%)	1 (1.4%)	1 (1.1%)	2 (0.9%)
	UK	2	0	1	1	0
Histology	IDC (tuble-forming type)	88 (11.8%)	39 (11.3%)	8 (11%)	23 (24%)	18 (7.7%)
	IDC (solid type)	179 (23.9%)	65 (18.8%)	19 (26%)	13 (13.5%)	82 (35.2%)
	IDC (scirrhous type)	321 (42.9%)	164 (47.4%)	33 (45.2%)	46 (47.9%)	78 (33.5%)
	IDC (special type)	32 (4.3%)	17 (4.9%)	2 (2.7%)	4 (4.2%)	9 (3.9%)
	IDC (UK)	128 (17.1%)	61 (17.6%)	11 (15.1%)	10 (10.4%)	46 (19.7%)
	UK	5	1	2	0	2
ER	Positive	430 (57.1%)	338 (97.4%)	0	92 (95.8%)	0
	Negative	323 (42.9%)	9 (2.6%)	75 (100%)	4 (4.2%)	235 (100%)
PgR	Positive	336 (44.7%)	273 (78.9%)	0	63 (65.6%)	0
	Negative	416 (55.3%)	73 (21.1%)	75 (100%)	33 (34.4%)	235 (100%)
	UK	1	1	0	0	0
HER2	Positive	171 (22.7%)	0	75 (100%)	96 (100%)	0
	Negative	582 (77.3%)	347 (100%)	0	0	235 (100%)
Nuclear grade	1	144 (24.4%)	92 (32.9%)	2 (4.5%)	11 (15.7%)	39 (19.9%)
	2	137 (23.2%)	80 (28.6%)	9 (20.5%)	22 (31.4%)	26 (13.3%)
	3	309 (52.4%)	108 (38.6%)	33 (75%)	37 (52.9%)	131 (66.8%)
	UK	163	67	31	26	39
Histological grade	1	55 (18.5%)	39 (22.8%)	1 (8.3%)	1 (25%)	14 (12.7%)
	2	109 (36.7%)	77 (45%)	3 (25%)	2 (50%)	27 (24.5%)
	3	133 (4.48%)	55 (32.2%)	8 (66.7%)	1 (25%)	69 (62.7%)
	UK	456	176	63	92	125
Ki-67	< 40%	264 (46.0%)	164 (58.4%)	17 (42.5%)	24 (42.9%)	59 (29.9%)
	≥ 40%	310 (54.0%)	117 (41.6%)	23 (57.5%)	32 (57.1%)	138 (70.1%)
	UK	179	66	35	40	38
Stage	I	47 (6.2%)	8 (2.3%)	6 (8%)	12 (12.5%)	21 (8.9%)
	IIA	260 (34.5%)	115 (33.1%)	16 (21.3%)	23 (24%)	106 (45.1%)
	IIB	279 (37.1%)	146 (42.1%)	28 (37.3%)	34 (35.4%)	71 (30.2%)
	IIIA	88 (11.7%)	43 (12.4%)	15 (20%)	12 (12.5%)	18 (7.7%)
	IIIB	35 (4.6%)	19 (5.5%)	3 (4%)	7 (7.3%)	6 (2.6%)
	IIIC	44 (5.8%)	16 (4.6%)	7 (9.3%)	8 (8.3%)	13 (5.5%)
Use of Trastuzumab	Yes	167 (22.2%)	1 (0.3%)	71 (95.9%)	95 (99%)	0
	No	584 (77.7%)	345 (99.7%)	3 (4.1%)	1 (1%)	235 (100%)
	UK	2	1	1	0	0
Total		753				

*SD* standard deviation, *UK* unknown

### pCR rates based on IPD

**Fig. 2:**
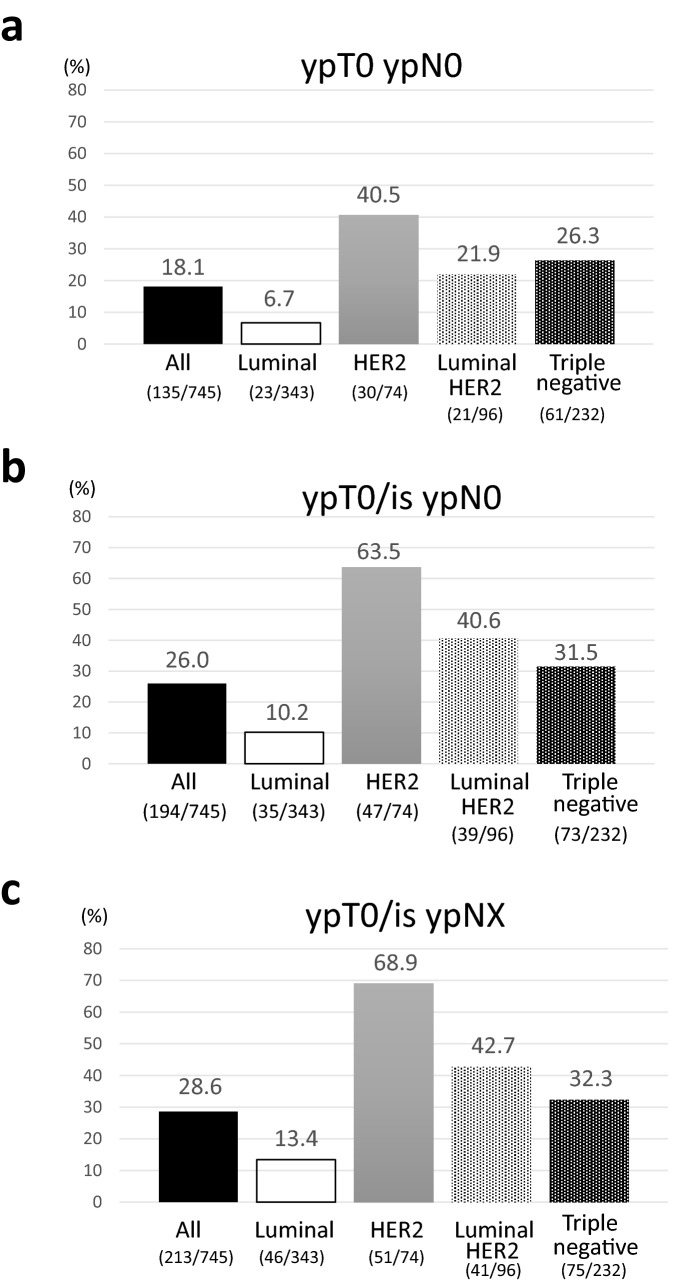
pCR rates in each breast cancer subtype. **a** ypT0 ypN0, **b** ypT0/is ypN0, **c** ypT0/is ypNX. The actual percentage is given above the corresponding bar

Among the efficacy analysis population (745 patients), surgery was performed in 743 patients. In each subtype, the three pCRs (ypT0 ypN0, ypT0/is ypN0, and ypT0/is ypNX) were observed in 6.7% (95% CI: 4.3–9.9), 10.2% (7.2–13.9), and 13.4% (10.0–17.5) of the patients for luminal; 40.5% (29.3–52,6), 63.5% (51.5–74.4), and 68.9% (57.1–79.2) of the patients for HER2-rich; 21.9% (14.1–31.5), 40.6% (30.7–51.1), and 42.7% (32.7–53.2) of the patients for luminal/HER2; and 26.3% (20.7–32.5), 31.5% (25.5–37.9), and 32.3% (26.4–38.8) of the patients for TNBC, respectively (Fig. 2). A forest plot of the proportion of ypT0 ypN0, ypT0/is ypN0, and ypT0/is ypNX patients according to subgroup variables is shown in Suppl. Fig. 3. In Suppl. Fig. 3b for the population of ypT0/is ypN0, the pCR rates for NG 1, 2, and 3 were 7.7% (95% CI: 3.9–13.3), 17.8% (11.7–25.3), and 34.6% (29.3–40.3); those for low (< 40%) and high (≥ 40%) Ki-67 expression were 12.5% (8.8–17.2) and 33.9% (28.6–39.5); those for cStages I, IIA, IIB, and IIIA were 37.0% (23.2–52.5), 31.4% (25.8–37.4), 23.0% (18.2–28.4), and 23.5% (15.0–34.0); and those for HER2-positive and -negative cases were 50.6% (42.8–58.3) and 18.8% (15.7–22.2), respectively. The other forest plots for ypT0 ypN0 and ypT0/is ypNX revealed results similar to those shown in Suppl. Fig. 3a, c.

### Clinical variables associated with pCR

The ORs of clinical variables are shown in Table 3. The univariate analysis showed that subtype, NG, HG, high Ki-67, early cStage, use of trastuzumab, and effect of NAC were significantly associated with each pCR rate. Menopause, HG, and the use of trastuzumab were not assessed using the multivariate model because of the collinearity between menopause and age, as well as HG, NG, HER2-positive status, and the use of trastuzumab. Clinical evaluation of NAC was not performed because it was a result of, rather than a risk factor for, the response. The ORs for the HER2-rich, luminal/HER2, and TNBC groups were 15.14 (7.81–29.33), 6.33 (3.50–11.45), and 2.97 (1.84–4.80), respectively. Those for NG 2 and 3 were 1.72 (0.79–3.73) and 2.90 (1.48–5.71) times higher than those for NG 1, and high Ki-67 had 2.51 (1.52–4.15) times higher ORs than low Ki-67. The OR for weekly nab-PTX was 2.14 (1.03–4.43) times higher than that for q3w nab-PTX. The ORs for ypT0 ypN0 and ypT0/is ypNX were similar to that for ypT0/is ypN0 (Suppl. Table 2a, b).

**Table 3:** Univariate and multivariate logistic regression analyses of pCR (ypT0is ypN0)

Variables	Subgroup	Univariable logistic model				Multivariable logistic model			
		OR	95% CI		*P* value	OR	95% CI		*P* value
Subtype	Luminal	Reference							
	HER2-rich	15.32	8.50	27.59	< 0.001	15.14	7.81	29.33	< 0.001
	Luminal/HER2	6.02	3.52	10.30	< 0.001	6.33	3.5	11.45	< 0.001
	Triple negative	4.04	2.59	6.31	< 0.001	2.97	1.84	4.80	< 0.001
Menopause	Post/pre	1.15	0.83	1.60	0.404				
Age	< 40	Reference							
	40– < 60	1.16	0.71	1.92	0.551	1.57	0.88	2.77	0.124
	60–	0.98	0.56	1.69	0.928	1.33	0.7	2.53	0.382
Nuclear grade	1	Reference							
	2	2.60	1.22	5.53	0.014	1.72	0.79	3.73	0.172
	3	6.36	3.29	12.29	< 0.001	2.90	1.48	5.71	0.002
Histological grade	1	Reference				–			
	2	7.47	0.95	58.67	0.056	–			
	3	25.20	3.37	188.37	0.002	–			
Ki-67	40% ≤ / < 40%	3.57	2.31	5.52	< 0.001	2.51	1.52	4.15	< 0.001
cStage	I	Reference				Reference			
	II	0.63	0.34	1.19	0.153	0.83	0.41	1.71	0.618
	III	0.42	0.20	0.85	0.016	0.47	0.21	1.06	0.067
Order of administering nab-PTX	After/Before A	1.40	0.88	2.22	0.156	0.76	0.32	1.78	0.522
Sequence of administering nab-PTX	Weekly/q3w	1.17	0.80	1.71	0.418	2.14	1.03	4.43	0.041
Use of Trastuzumab		4.67	3.23	6.75	< 0.001	–			
Clinical evaluation of NAC	cCR	Reference							
	PR	0.08	0.05	0.11	< 0.001				
	SD	0.02	0.01	0.06	< 0.001				
	PD	0.02	0.00	0.11	< 0.001				

*cCR* clinical complete response, *PR* partial response, *SD* stable disease, *PD* progressive disease *After/Before A* After/Before anthracycline

### Toxicity profiling

The AEs ≥ G3 were as follows: neutropenia, 39.7%; leukopenia, 22.5%; peripheral sensory neuropathy, 9.7%; febrile neutropenia (FN), 9.6%; myalgia, 5.7%; hepatobiliary disorders, 5.5%; and arthralgia, 4.8% (Table 4). The AEs were different between q3w and weekly nab-PTX. Neutropenia (19.9 vs. 48.0%, *p* < 0.0001), leukopenia (18.5 vs. 35.8%, *p* < 0.0001), peripheral sensory neuropathy (7.3 vs. 17.9%, *p* = 0.0304), myalgia (3.3 vs. 14.0%, *p* < 0.0001), arthralgia (2.3 vs. 13.5%, *p* < 0.0001), and vomiting (3.1 vs. 7.5%, *p* = 0.0304) were less frequent in the q3w group than in the weekly group, respectively. However, hepatobiliary disorder was observed more frequently in the q3w group than in the weekly group (6.4 vs. 2.3%, *p* = 0.0304). Only one patient died of febrile neutropenia during the 5-fluorouracil, epirubicin, and cyclophosphamide (FEC) treatment.

**Table 4:** Adverse events (≥ grade 3) depending on therapy schedule

Adverse event	Incidence						Chi-squre test
	Total		q3w		Weekly		
	Number	%	Number	%	Number	%	*p* value
Neutropenia	298/751	39.7	115/578	19.9	83/173	48.0	< 0.0001
Leukopenia	169/750	22.5	107/577	18.5	62/173	35.8	< 0.0001
Peripheral sensory neuropathy	73/751	9.7	42/578	7.3	31/173	17.9	< 0.0001
Febrile neutropenia	72/751	9.6	61/578	10.6	11/173	6.4	*p* = 0.1
Myalgia	43/751	5.7	19/578	3.3	24/171	14.0	< 0.0001
Hepatobiliary disorders	41/751	5.5	37/578	6.4	4/173	2.3	0.0378
Arthralgia	36/749	4.8	13/578	2.3	23/171	13.5	< 0.0001
Vomitng	31/751	4.1	18/578	3.1	13/173	7.5	0.0304
Peripheral motor neuropathy	17/751	2.3	16/578	2.9	1/173	0.1	0.0893
Infusion reaction	6/751	0.8	4/578	0.7	2/173	1.2	0.5475
Cardiac disorders	4/751	0.5	2/578	0.4	2/173	1.2	0.1991
Death	1/753	0.1	1/580	0.2	0/173	0.0	NA

### Dose of nab-PTX in drug sequence

The total dose of nab-PTX administered to the patients is presented in Suppl. Table 3. The mean dose ± standard deviation for all patients was 1060.6 ± 237.9 mg. The total q3w and weekly doses were 1004.3 ± 116.7 mg and 1263.9 ± 358.0 mg, respectively (*p* < 0.0001). Regarding drug sequence, the total dose for anthracycline followed by nab-PTX was 1052.3 ± 209.4 mg (q3w: 1003.7 ± 117.5 mg, weekly: 1372.4 ± 353.0 mg; *p* < 0.0001), and that for nab-PTX followed by anthracycline was 1114.0 ± 369.8 mg (q3w: 1040 mg, weekly: 1159.0 ± 332.3 mg; *p* = 0.2875).

### Prognosis for patients with pCR (ypT0/is ypN0) treated with nab-PTX-containing regimens

**Fig. 3:**
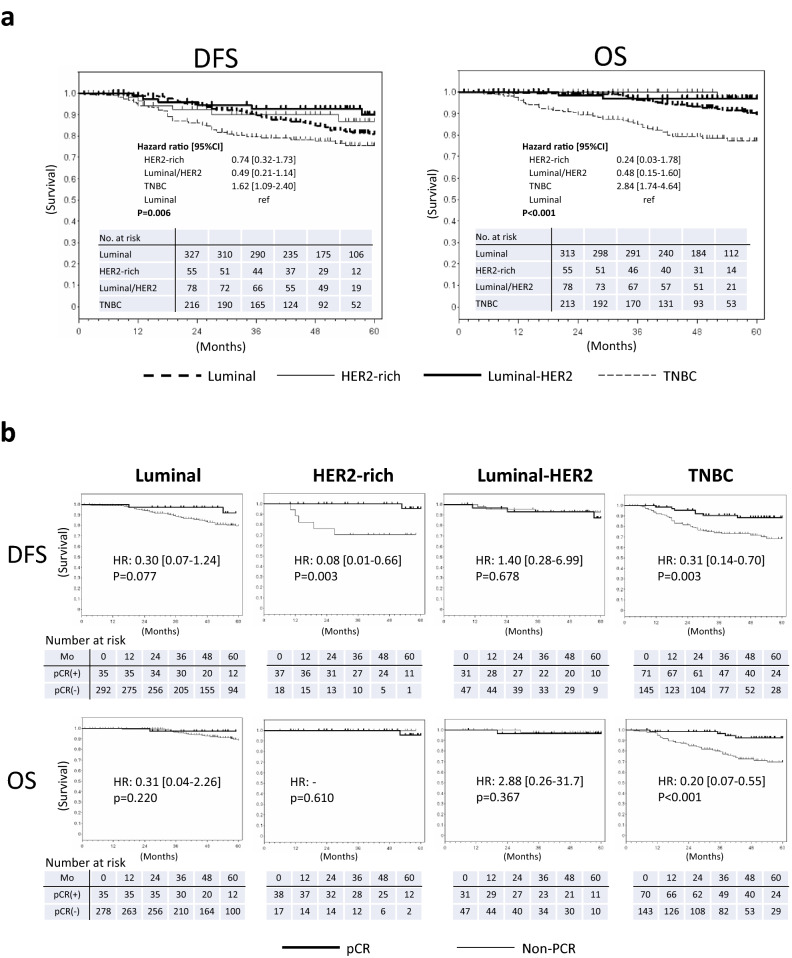
Kaplan–Meier curves estimates for DFS and OS. **a** Kaplan–Meier estimates stratified by subtype. DFS (left) and OS (right) are shown with hazard ratios (HRs). **b** Kaplan–Meier estimates stratified by pCR (ypT0/is ypN0). Survival comparison between pCR and non-PCR populations is indicated for DFS (upper panels) and OS (lower panels) in each subtype. The HR and *p* value are indicated in each graph

The Kaplan–Meier curves of DFS and OS are shown in Fig. 3a. The DFS rates at 5 years were 80.7, 86.9, 90.0, and 75.5% for luminal, HER2-rich, luminal/HER2, and TNBC subtypes, respectively (Fig. 3a, left panel). The DFS rates stratified by pCR (ypT0/is ypN0) are shown in Fig. 3b (upper panel). In the HER2-rich and TNBC subtypes, the DFS for patients with pCR (ypT0/is ypN0) was significantly longer than that for patients without pCR. The OS rates at 5 years were 89.6, 96.6, 97.1, and 77.4% for luminal, HER2-rich, luminal/HER2, and TNBC subtypes, respectively (Fig. 3a, right panel). The OS rates stratified by pCR (ypT0/is ypN0) are shown in Fig. 3b (lower panel). The OS for patients in TNBC was significantly longer with pCR than without pCR. However, in the luminal and luminal/HER2 subtypes, there was no statistical difference between the groups. The prognoses for pCR (ypT0 ypN0 and ypT0/is ypNX) were similar to those for pCR (ypT0/is ypN0) (Suppl. Fig. 4a, b).

## Discussion

We analyzed the pCR rates using IPD data based on three pCR definitions [16]. Nodal involvement after NAC was associated with an increased risk of tumor recurrence and death, and a preferable prognosis was not associated with axillary residual tumors or intraductal tumors in the breast. Therefore, we recognize that ypT0 ypN0 and ypT0/is ypN0 are clinically useful pCRs, particularly in patients with aggressive phenotypes such as HER2-rich or TNBC subtypes [22–24]. Our results indicated that the pCR rates of luminal-type tumors were 6.7, 10.2, and 13.4%, respectively, which are similar to the findings for anthracycline and taxane chemotherapy, supporting the power of nab-PTX for ER-positive subtypes [25]. Although the multivariate analysis demonstrated that TNBC is statistically associated with nab-PTX-related pCR (OR: 2.97), the pCR rate (31.5% for ypT0/is ypN0) was similar to previous findings for anthracycline and taxane chemotherapy [16, 22, 23]. However, recent studies have demonstrated that weekly nab-PTX administration induced a high pCR rate (41–49%) [26–28]. In our study, the pCR rates for TNBC were 41.7% (20/48) by weekly nab-PTX and 28.8% (53/184) by q3w, respectively. The total dose of nab-PTX was higher with weekly administration than with q3w (Suppl. Table 3). Our findings might have been affected by the higher proportion of patients treated with q3w nab-PTX (83%), resulting in a low pCR rate. Recent publications suggest new strategies, including dose-dense chemotherapy, platinum-containing regimens, or combinations with molecular-targeted agents lead to better results, showing ≥ 50% pCR rates [29–32]. It should be noted that TNBC-specific characteristics, including high NG, high Ki-67, and PD-L1 expression may have affected these results.

We hypothesized that nab-PTX would have a substantial effect on HER2-positive BC, and we subsequently found that the pCR (ypT0/is ypN0) rates were 63.5% for HER2-rich and 40.6% for luminal/HER2 subtypes. As reported in the NOAH trial and GeparQuattro study, the HER2-positive subtype showed good responses to anthracycline followed by taxane with trastuzumab, with pCR rates of 38–43.5% [33, 34]. The pooled analysis indicated an additional power of 30–50% using trastuzumab [23]. In the NeoALTTO study, the pCR rate of the HER2-rich subtype reached 61.3% after treatment with paclitaxel and dual blockage using trastuzumab and lapatinib [35]. The NeoSphere trial also reported that the pCR rate was 63.2% after treatment with docetaxel combined with trastuzumab and pertuzumab [36]. In our study, the pCR rates reached 63.5% (ypT0/is ypN0) for the HER2-rich subtype with nab-PTX and trastuzumab, and this was similar to the value obtained with a dual anti-HER2 blockage regimen. In the GeparSepto trial, the combination of nab-PTX and dual blockage using pertuzumab and trastuzumab demonstrated remarkable results, showing that the pCR (ypT0 ypN0 and ypT0/is ypN0) rates reached 74.6 and 81.4%, respectively, for the HER2-rich subtype [28]. We obtained results similar to those with the combination of docetaxel/paclitaxel and dual HER2 blockage described above. Despite no statistically significant difference, the pCR (ypT0/is ypN0) rates for the HER2-rich subtype were 59.7% (q3w) and 76.5% (weekly). The ORs for the HER2-rich and luminal/HER2 subtypes in the multivariate analysis were 15.14 and 6.33, respectively, which reflect the cases with high NG and/or Ki-67 expression, as shown in Table 2.

As shown in Table 4, our AE data were obtained using both nab-PTX and anthracycline. Hematological toxicities are reportedly common for both drugs [6, 26–28]. These results suggest that the toxicities of neoadjuvant nab-PTX are tolerable. Weekly nab-PTX produced more frequent and severe AEs than q3w nab-PTX for the following three reasons. (1) The total dose with weekly administration was considerably higher than that with q3w regimen. (2) Weekly administration resulted in more frequent hospital visits to observe AEs than q3w administration. (3) Our data were obtained from IPD with almost no missing data points, which enabled us to perform precise analyses.

Patients with pCR showed a better prognosis in all subtypes. In particular, the DFS in both HER2-rich and TNBC subtypes was longer with pCR than without pCR. Only TNBC patients with pCR were associated with an improved OS. Our study showed no difference in OS between pCR and non-PCR groups with the HER2-rich subtype, as observed in the NeoSphere study [37]. Although the NeoALLTO study showed a significant association between pCR and both DFS and OS, the OS of HER2-positive BC patients after NAC remains unclear because newly developed anti-HER2 therapies for metastatic BC may strongly affect long-term survival [38]. Our data demonstrated that nab-PTX for NAC induced higher pCR rate particularly in HER2-positive BC patients by combination with trastuzumab. These results may modify the adjuvant therapy against primary HER2-positive BC. If pathologically negative lymph node is found after surgery, trastuzumab monotherapy may be enough in the adjuvant setting. However, in cases of positive lymph node or residual invasive disease, escalating therapies using either pertuzumab or trastuzumab emtansine (T-DM1) may be employed [39, 40]. Patients with pCR who present long DFS could de-escalate the additional anti-HER2 therapies in the adjuvant setting and delay those in the metastatic setting. Although HER2-positive MBC may be sensitive to anti-HER2 therapy, it is the best drug for patients with primary breast cancer.

In this study, we assessed the risk of bias using RoBANS and classified it as moderate [19, 20]. As most of the studies in this meta-analysis were not RCTs, the moderate risk overall does not indicate low study quality. The heterogeneity among the 16 studies was moderate (*I*^*2*^ = 68.8%). Unlike an RCT, the summary measure of a single-arm study directly reflects the distribution of the baseline characteristics. Actually, two studies showed low pCR rates in patients with luminal or TNBC subtypes, and high proportions of patients with cStage III. The existence of moderate heterogeneity indicates the need for IPD analysis to minimize heterogeneity.

In conclusion, nab-PTX is an acceptable chemotherapeutic agent for aggressive breast cancers such as HER2-rich, luminal/HER2, and TNBC subtypes in a neoadjuvant setting. Nab-PTX monotherapy is a useful option for TNBC. The combination of nab-PTX and anti-HER2 can achieve desirable pCR rates in patients with HER2-positive subtypes and manageable toxicity. Antibody drug conjugates, such as T-DM1 and trastuzumab deruxtecan (T-Dxd), are being developed in this field. Furthermore, trastuzumab and pertuzumab combined with taxane, and anti-microtubule agents are still considered standard preoperative or adjuvant therapy. Although there is a need for further clinical studies to replace the conventional docetaxel or paclitaxel with nab-PTX, nab-PTX will be considered as a potential chemotherapeutic agent in combination with anti-HER2 antibodies to enhance their efficacy.

## Supplementary Information

Below is the link to the electronic supplementary material.

Supplementary file1 (XLSX 24 KB)

Supplementary file2 (PPTX 383 KB)
