# Supplementary material for: Meta-analysis of nanoparticle albumin-bound paclitaxel used as neoadjuvant chemotherapy for operable breast cancer based on individual patient data (JBCRG-S01 study)
Source: Breast Cancer. 2021 Apr 3;28(5):1023–37. doi: 10.1007/s12282-021-01238-9 (PMC8354972; doi:10.1007/s12282-021-01238-9)
Supplement: Supplementary file 2 — Supplementary file2 (PPTX 383 KB) [file 12282_2021_1238_MOESM2_ESM.pptx]

## Slide 1
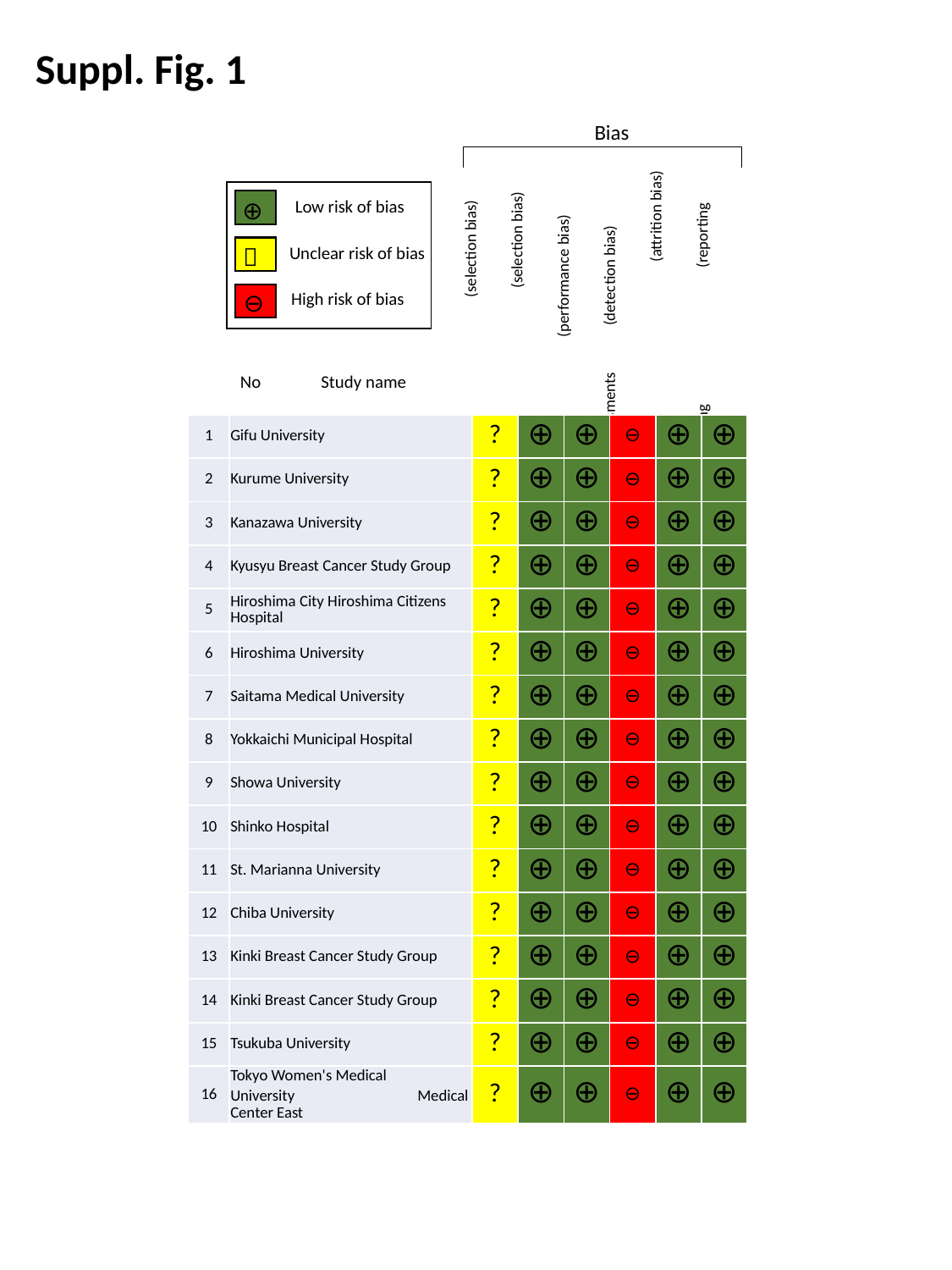

Suppl. Fig. 1
Bias
| Selection of participants (selection bias) | Confounding variables (selection bias) | Measurement of exposure (performance bias) | Blinding of outcome assessments (detection bias) | Incomplete outcome data (attrition bias) | Selective outcome reporting (reporting bias) |
| --- | --- | --- | --- | --- | --- |
⊕
Low risk of bias
？
Unclear risk of bias
High risk of bias
⊖
No Study name
| 1 | Gifu University | ? | ⊕ | ⊕ | ⊖ | ⊕ | ⊕ |
| --- | --- | --- | --- | --- | --- | --- | --- |
| 2 | Kurume University | ? | ⊕ | ⊕ | ⊖ | ⊕ | ⊕ |
| 3 | Kanazawa University | ? | ⊕ | ⊕ | ⊖ | ⊕ | ⊕ |
| 4 | Kyusyu Breast Cancer Study Group | ? | ⊕ | ⊕ | ⊖ | ⊕ | ⊕ |
| 5 | Hiroshima City Hiroshima Citizens Hospital | ? | ⊕ | ⊕ | ⊖ | ⊕ | ⊕ |
| 6 | Hiroshima University | ? | ⊕ | ⊕ | ⊖ | ⊕ | ⊕ |
| 7 | Saitama Medical University | ? | ⊕ | ⊕ | ⊖ | ⊕ | ⊕ |
| 8 | Yokkaichi Municipal Hospital | ? | ⊕ | ⊕ | ⊖ | ⊕ | ⊕ |
| 9 | Showa University | ? | ⊕ | ⊕ | ⊖ | ⊕ | ⊕ |
| 10 | Shinko Hospital | ? | ⊕ | ⊕ | ⊖ | ⊕ | ⊕ |
| 11 | St. Marianna University | ? | ⊕ | ⊕ | ⊖ | ⊕ | ⊕ |
| 12 | Chiba University | ? | ⊕ | ⊕ | ⊖ | ⊕ | ⊕ |
| 13 | Kinki Breast Cancer Study Group | ? | ⊕ | ⊕ | ⊖ | ⊕ | ⊕ |
| 14 | Kinki Breast Cancer Study Group | ? | ⊕ | ⊕ | ⊖ | ⊕ | ⊕ |
| 15 | Tsukuba University | ? | ⊕ | ⊕ | ⊖ | ⊕ | ⊕ |
| 16 | Tokyo Women's Medical University　　　　　　　 Medical Center East | ? | ⊕ | ⊕ | ⊖ | ⊕ | ⊕ |

## Slide 2
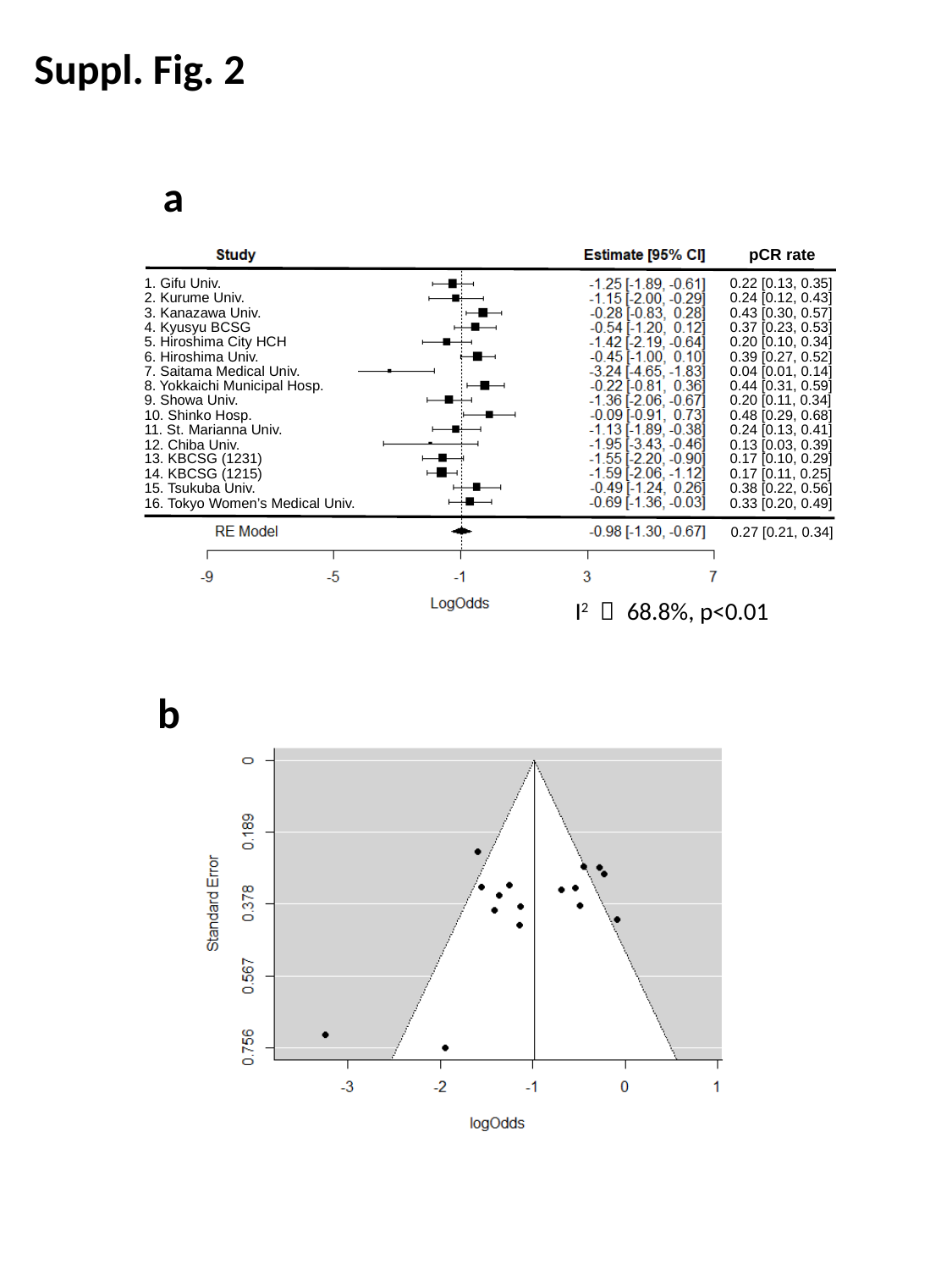

Suppl. Fig. 2
a
pCR rate
| 1. Gifu Univ. |
| --- |
| 2. Kurume Univ. |
| 3. Kanazawa Univ. |
| 4. Kyusyu BCSG |
| 5. Hiroshima City HCH |
| 6. Hiroshima Univ. |
| 7. Saitama Medical Univ. |
| 8. Yokkaichi Municipal Hosp. |
| 9. Showa Univ. |
| 10. Shinko Hosp. |
| 11. St. Marianna Univ. |
| 12. Chiba Univ. |
| 13. KBCSG (1231) |
| 14. KBCSG (1215) |
| 15. Tsukuba Univ. |
| 16. Tokyo Women’s Medical Univ. |
| 0.22 [0.13, 0.35] |
| --- |
| 0.24 [0.12, 0.43] |
| 0.43 [0.30, 0.57] |
| 0.37 [0.23, 0.53] |
| 0.20 [0.10, 0.34] |
| 0.39 [0.27, 0.52] |
| 0.04 [0.01, 0.14] |
| 0.44 [0.31, 0.59] |
| 0.20 [0.11, 0.34] |
| 0.48 [0.29, 0.68] |
| 0.24 [0.13, 0.41] |
| 0.13 [0.03, 0.39] |
| 0.17 [0.10, 0.29] |
| 0.17 [0.11, 0.25] |
| 0.38 [0.22, 0.56] |
| 0.33 [0.20, 0.49] |
0.27 [0.21, 0.34]
 I2 ＝ 68.8%, p<0.01
b

## Slide 3
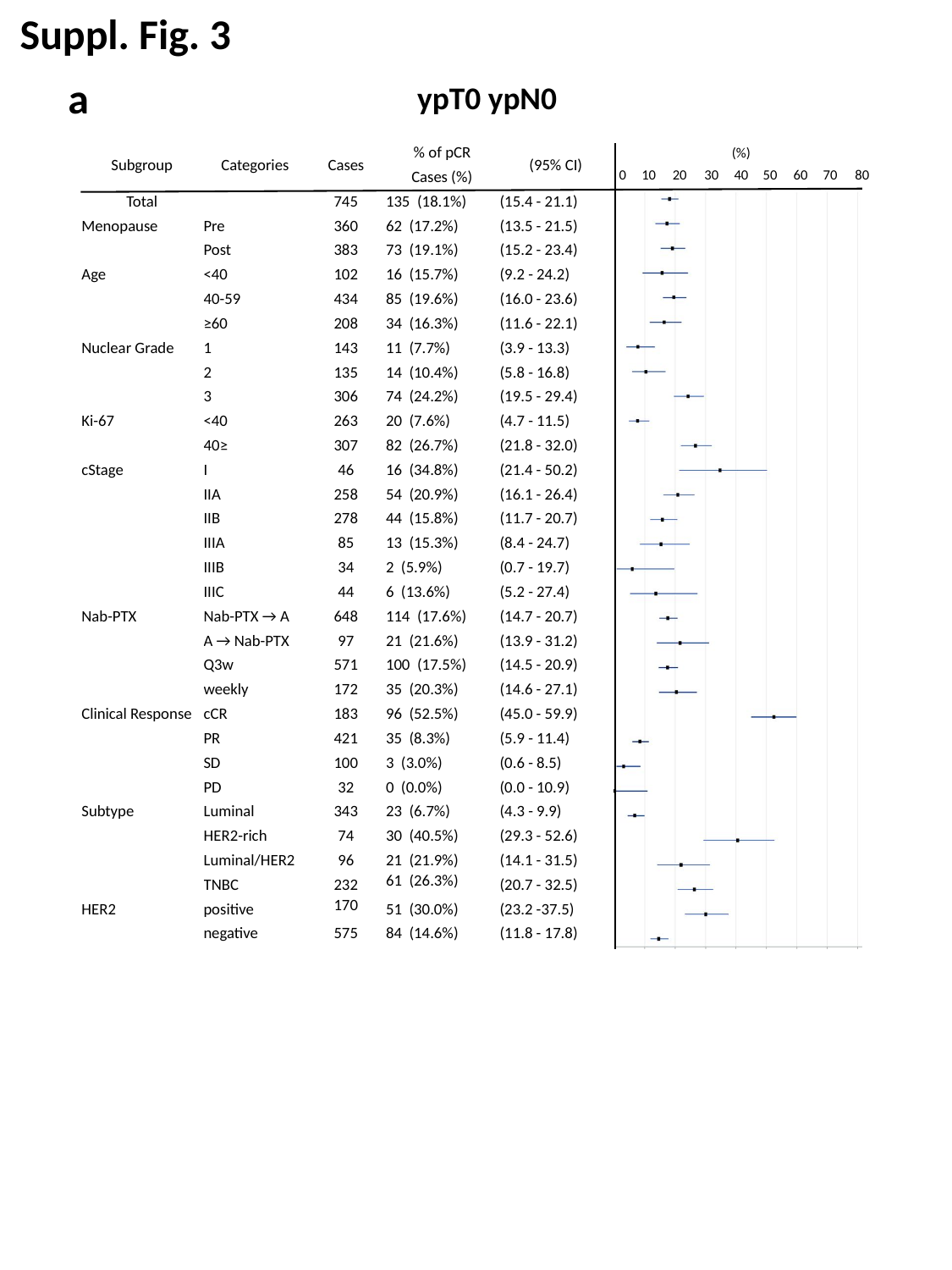

Suppl. Fig. 3
a
ypT0 ypN0
(%)
60
50
0
10
20
30
40
70
80
| Subgroup | Categories | Cases | % of pCR | (95% CI) |
| --- | --- | --- | --- | --- |
| | | | Cases (%) | |
| Total | | 745 | 135 (18.1%) | (15.4 - 21.1) |
| Menopause | Pre | 360 | 62 (17.2%) | (13.5 - 21.5) |
| | Post | 383 | 73 (19.1%) | (15.2 - 23.4) |
| Age | <40 | 102 | 16 (15.7%) | (9.2 - 24.2) |
| | 40-59 | 434 | 85 (19.6%) | (16.0 - 23.6) |
| | ≥60 | 208 | 34 (16.3%) | (11.6 - 22.1) |
| Nuclear Grade | 1 | 143 | 11 (7.7%) | (3.9 - 13.3) |
| | 2 | 135 | 14 (10.4%) | (5.8 - 16.8) |
| | 3 | 306 | 74 (24.2%) | (19.5 - 29.4) |
| Ki-67 | <40 | 263 | 20 (7.6%) | (4.7 - 11.5) |
| | 40≥ | 307 | 82 (26.7%) | (21.8 - 32.0) |
| cStage | I | 46 | 16 (34.8%) | (21.4 - 50.2) |
| | IIA | 258 | 54 (20.9%) | (16.1 - 26.4) |
| | IIB | 278 | 44 (15.8%) | (11.7 - 20.7) |
| | IIIA | 85 | 13 (15.3%) | (8.4 - 24.7) |
| | IIIB | 34 | 2 (5.9%) | (0.7 - 19.7) |
| | IIIC | 44 | 6 (13.6%) | (5.2 - 27.4) |
| Nab-PTX | Nab-PTX → A | 648 | 114 (17.6%) | (14.7 - 20.7) |
| | A → Nab-PTX | 97 | 21 (21.6%) | (13.9 - 31.2) |
| | Q3w | 571 | 100 (17.5%) | (14.5 - 20.9) |
| | weekly | 172 | 35 (20.3%) | (14.6 - 27.1) |
| Clinical Response | cCR | 183 | 96 (52.5%) | (45.0 - 59.9) |
| | PR | 421 | 35 (8.3%) | (5.9 - 11.4) |
| | SD | 100 | 3 (3.0%) | (0.6 - 8.5) |
| | PD | 32 | 0 (0.0%) | (0.0 - 10.9) |
| Subtype | Luminal | 343 | 23 (6.7%) | (4.3 - 9.9) |
| | HER2-rich | 74 | 30 (40.5%) | (29.3 - 52.6) |
| | Luminal/HER2 | 96 | 21 (21.9%) | (14.1 - 31.5) |
| | TNBC | 232 | 61 (26.3%) | (20.7 - 32.5) |
| HER2 | positive | 170 | 51 (30.0%) | (23.2 -37.5) |
| | negative | 575 | 84 (14.6%) | (11.8 - 17.8) |

## Slide 4
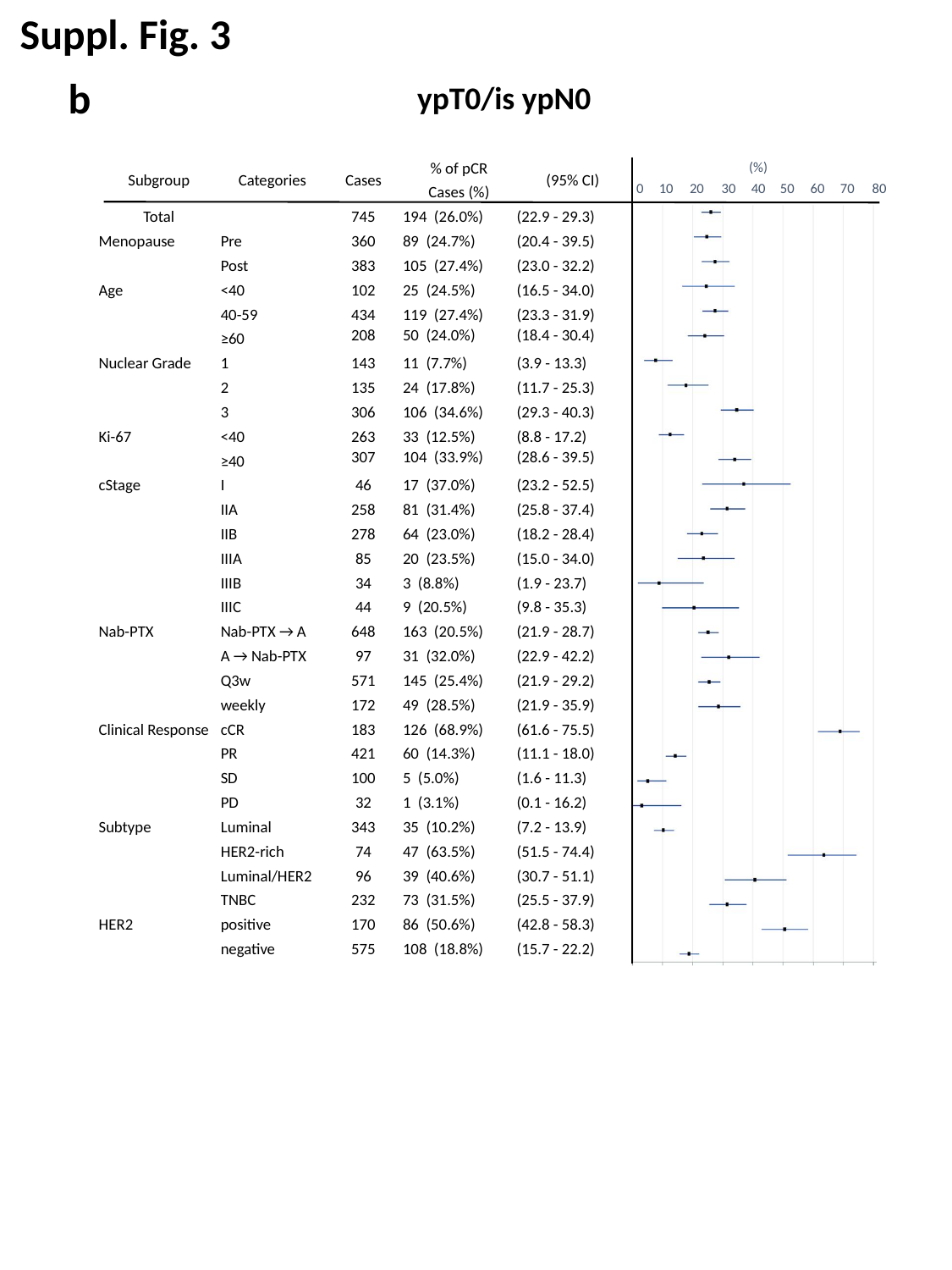

Suppl. Fig. 3
b
ypT0/is ypN0
(%)
60
50
0
10
20
30
40
70
80
| Subgroup | Categories | Cases | % of pCR | (95% CI) |
| --- | --- | --- | --- | --- |
| | | | Cases (%) | |
| Total | | 745 | 194 (26.0%) | (22.9 - 29.3) |
| Menopause | Pre | 360 | 89 (24.7%) | (20.4 - 39.5) |
| | Post | 383 | 105 (27.4%) | (23.0 - 32.2) |
| Age | <40 | 102 | 25 (24.5%) | (16.5 - 34.0) |
| | 40-59 | 434 | 119 (27.4%) | (23.3 - 31.9) |
| | ≥60 | 208 | 50 (24.0%) | (18.4 - 30.4) |
| Nuclear Grade | 1 | 143 | 11 (7.7%) | (3.9 - 13.3) |
| | 2 | 135 | 24 (17.8%) | (11.7 - 25.3) |
| | 3 | 306 | 106 (34.6%) | (29.3 - 40.3) |
| Ki-67 | <40 | 263 | 33 (12.5%) | (8.8 - 17.2) |
| | ≥40 | 307 | 104 (33.9%) | (28.6 - 39.5) |
| cStage | I | 46 | 17 (37.0%) | (23.2 - 52.5) |
| | IIA | 258 | 81 (31.4%) | (25.8 - 37.4) |
| | IIB | 278 | 64 (23.0%) | (18.2 - 28.4) |
| | IIIA | 85 | 20 (23.5%) | (15.0 - 34.0) |
| | IIIB | 34 | 3 (8.8%) | (1.9 - 23.7) |
| | IIIC | 44 | 9 (20.5%) | (9.8 - 35.3) |
| Nab-PTX | Nab-PTX → A | 648 | 163 (20.5%) | (21.9 - 28.7) |
| | A → Nab-PTX | 97 | 31 (32.0%) | (22.9 - 42.2) |
| | Q3w | 571 | 145 (25.4%) | (21.9 - 29.2) |
| | weekly | 172 | 49 (28.5%) | (21.9 - 35.9) |
| Clinical Response | cCR | 183 | 126 (68.9%) | (61.6 - 75.5) |
| | PR | 421 | 60 (14.3%) | (11.1 - 18.0) |
| | SD | 100 | 5 (5.0%) | (1.6 - 11.3) |
| | PD | 32 | 1 (3.1%) | (0.1 - 16.2) |
| Subtype | Luminal | 343 | 35 (10.2%) | (7.2 - 13.9) |
| | HER2-rich | 74 | 47 (63.5%) | (51.5 - 74.4) |
| | Luminal/HER2 | 96 | 39 (40.6%) | (30.7 - 51.1) |
| | TNBC | 232 | 73 (31.5%) | (25.5 - 37.9) |
| HER2 | positive | 170 | 86 (50.6%) | (42.8 - 58.3) |
| | negative | 575 | 108 (18.8%) | (15.7 - 22.2) |

## Slide 5
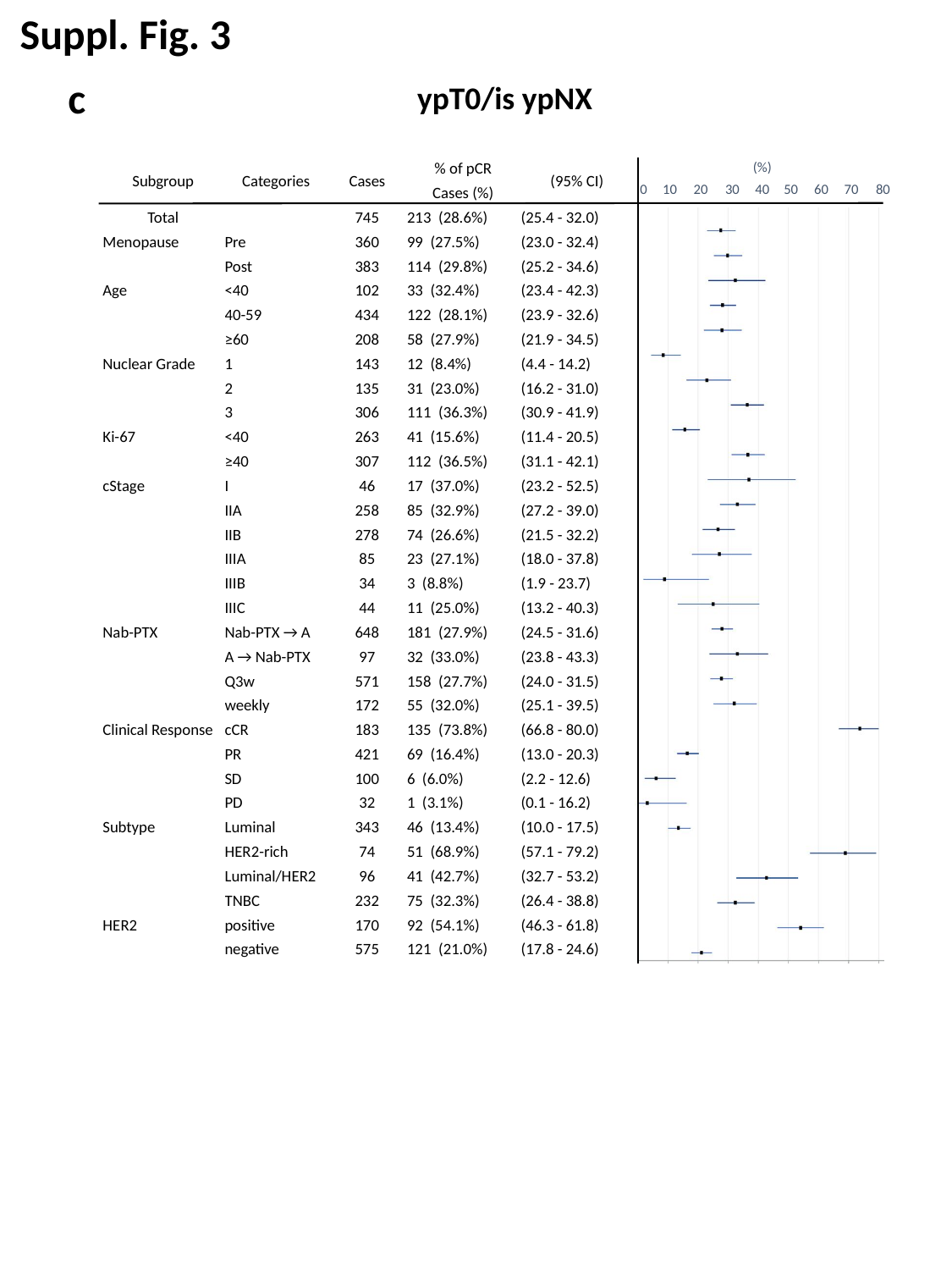

Suppl. Fig. 3
c
ypT0/is ypNX
(%)
60
50
0
10
20
30
40
70
80
| Subgroup | Categories | Cases | % of pCR | (95% CI) |
| --- | --- | --- | --- | --- |
| | | | Cases (%) | |
| Total | | 745 | 213 (28.6%) | (25.4 - 32.0) |
| Menopause | Pre | 360 | 99 (27.5%) | (23.0 - 32.4) |
| | Post | 383 | 114 (29.8%) | (25.2 - 34.6) |
| Age | <40 | 102 | 33 (32.4%) | (23.4 - 42.3) |
| | 40-59 | 434 | 122 (28.1%) | (23.9 - 32.6) |
| | ≥60 | 208 | 58 (27.9%) | (21.9 - 34.5) |
| Nuclear Grade | 1 | 143 | 12 (8.4%) | (4.4 - 14.2) |
| | 2 | 135 | 31 (23.0%) | (16.2 - 31.0) |
| | 3 | 306 | 111 (36.3%) | (30.9 - 41.9) |
| Ki-67 | <40 | 263 | 41 (15.6%) | (11.4 - 20.5) |
| | ≥40 | 307 | 112 (36.5%) | (31.1 - 42.1) |
| cStage | I | 46 | 17 (37.0%) | (23.2 - 52.5) |
| | IIA | 258 | 85 (32.9%) | (27.2 - 39.0) |
| | IIB | 278 | 74 (26.6%) | (21.5 - 32.2) |
| | IIIA | 85 | 23 (27.1%) | (18.0 - 37.8) |
| | IIIB | 34 | 3 (8.8%) | (1.9 - 23.7) |
| | IIIC | 44 | 11 (25.0%) | (13.2 - 40.3) |
| Nab-PTX | Nab-PTX → A | 648 | 181 (27.9%) | (24.5 - 31.6) |
| | A → Nab-PTX | 97 | 32 (33.0%) | (23.8 - 43.3) |
| | Q3w | 571 | 158 (27.7%) | (24.0 - 31.5) |
| | weekly | 172 | 55 (32.0%) | (25.1 - 39.5) |
| Clinical Response | cCR | 183 | 135 (73.8%) | (66.8 - 80.0) |
| | PR | 421 | 69 (16.4%) | (13.0 - 20.3) |
| | SD | 100 | 6 (6.0%) | (2.2 - 12.6) |
| | PD | 32 | 1 (3.1%) | (0.1 - 16.2) |
| Subtype | Luminal | 343 | 46 (13.4%) | (10.0 - 17.5) |
| | HER2-rich | 74 | 51 (68.9%) | (57.1 - 79.2) |
| | Luminal/HER2 | 96 | 41 (42.7%) | (32.7 - 53.2) |
| | TNBC | 232 | 75 (32.3%) | (26.4 - 38.8) |
| HER2 | positive | 170 | 92 (54.1%) | (46.3 - 61.8) |
| | negative | 575 | 121 (21.0%) | (17.8 - 24.6) |

## Slide 6
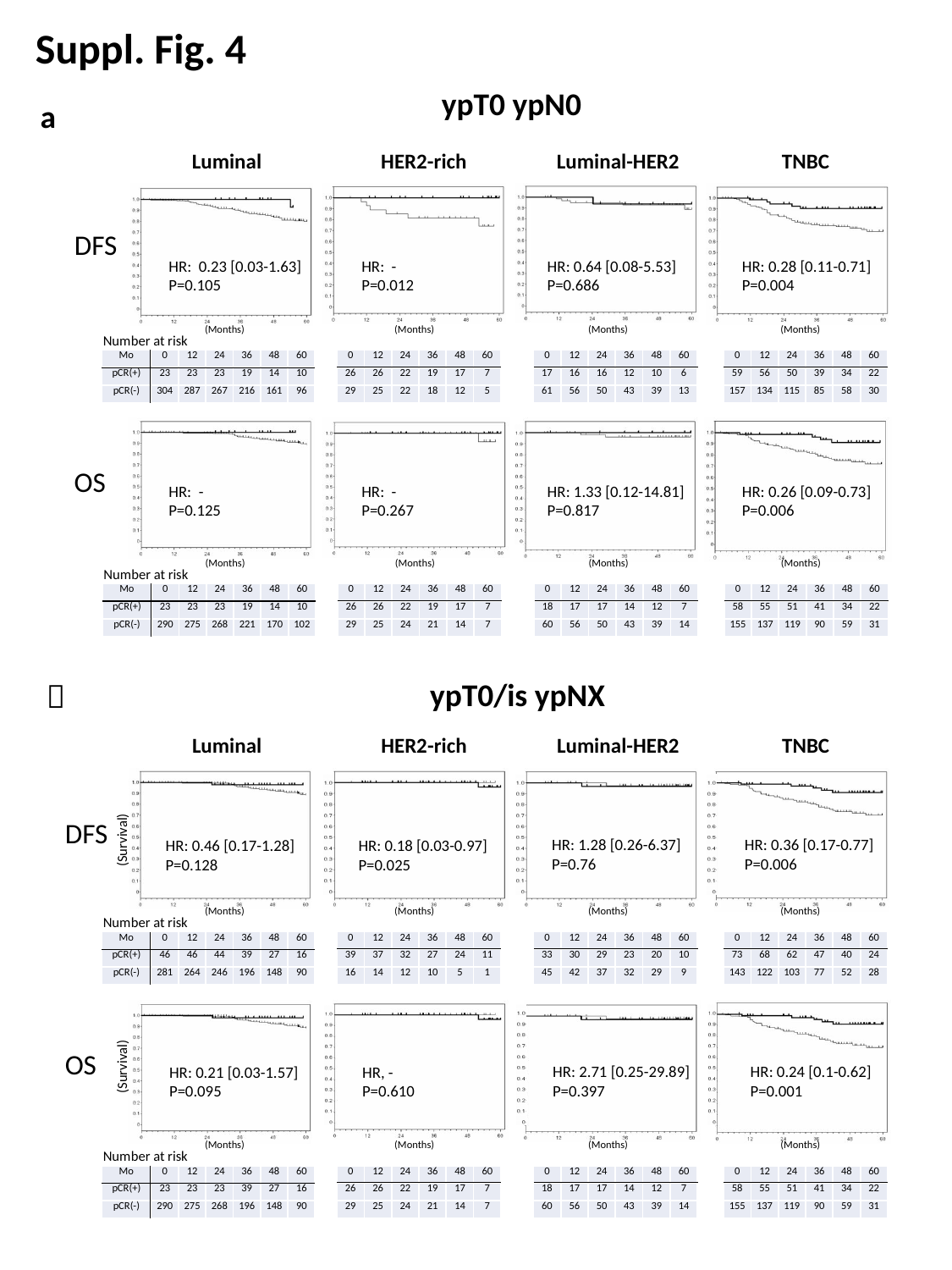

Suppl. Fig. 4
ypT0 ypN0
a
Luminal
HER2-rich
Luminal-HER2
TNBC
DFS
HR: 0.23 [0.03-1.63]
P=0.105
HR: -
P=0.012
HR: 0.64 [0.08-5.53]
P=0.686
HR: 0.28 [0.11-0.71]
P=0.004
| (Months) | |
| --- | --- |
| | |
| (Months) | |
| --- | --- |
| | |
| (Months) | |
| --- | --- |
| | |
| (Months) | |
| --- | --- |
| | |
Number at risk
| Mo |
| --- |
| pCR(+) |
| pCR(-) |
| 0 | 12 | 24 | 36 | 48 | 60 |
| --- | --- | --- | --- | --- | --- |
| 23 | 23 | 23 | 19 | 14 | 10 |
| 304 | 287 | 267 | 216 | 161 | 96 |
| 0 | 12 | 24 | 36 | 48 | 60 |
| --- | --- | --- | --- | --- | --- |
| 26 | 26 | 22 | 19 | 17 | 7 |
| 29 | 25 | 22 | 18 | 12 | 5 |
| 0 | 12 | 24 | 36 | 48 | 60 |
| --- | --- | --- | --- | --- | --- |
| 17 | 16 | 16 | 12 | 10 | 6 |
| 61 | 56 | 50 | 43 | 39 | 13 |
| 0 | 12 | 24 | 36 | 48 | 60 |
| --- | --- | --- | --- | --- | --- |
| 59 | 56 | 50 | 39 | 34 | 22 |
| 157 | 134 | 115 | 85 | 58 | 30 |
OS
HR: -
P=0.125
HR: -
P=0.267
HR: 1.33 [0.12-14.81]
P=0.817
HR: 0.26 [0.09-0.73]
P=0.006
| (Months) | |
| --- | --- |
| | |
| (Months) | |
| --- | --- |
| | |
| (Months) | |
| --- | --- |
| | |
| (Months) | |
| --- | --- |
| | |
Number at risk
| Mo |
| --- |
| pCR(+) |
| pCR(-) |
| 0 | 12 | 24 | 36 | 48 | 60 |
| --- | --- | --- | --- | --- | --- |
| 23 | 23 | 23 | 19 | 14 | 10 |
| 290 | 275 | 268 | 221 | 170 | 102 |
| 0 | 12 | 24 | 36 | 48 | 60 |
| --- | --- | --- | --- | --- | --- |
| 26 | 26 | 22 | 19 | 17 | 7 |
| 29 | 25 | 24 | 21 | 14 | 7 |
| 0 | 12 | 24 | 36 | 48 | 60 |
| --- | --- | --- | --- | --- | --- |
| 18 | 17 | 17 | 14 | 12 | 7 |
| 60 | 56 | 50 | 43 | 39 | 14 |
| 0 | 12 | 24 | 36 | 48 | 60 |
| --- | --- | --- | --- | --- | --- |
| 58 | 55 | 51 | 41 | 34 | 22 |
| 155 | 137 | 119 | 90 | 59 | 31 |
ypT0/is ypNX
ｂ
Luminal
HER2-rich
Luminal-HER2
TNBC
DFS
(Survival)
HR: 1.28 [0.26-6.37]
P=0.76
HR: 0.36 [0.17-0.77]
P=0.006
HR: 0.46 [0.17-1.28]
P=0.128
HR: 0.18 [0.03-0.97]
P=0.025
| (Months) | |
| --- | --- |
| | |
| (Months) | |
| --- | --- |
| | |
| (Months) | |
| --- | --- |
| | |
| (Months) | |
| --- | --- |
| | |
Number at risk
| Mo |
| --- |
| pCR(+) |
| pCR(-) |
| 0 | 12 | 24 | 36 | 48 | 60 |
| --- | --- | --- | --- | --- | --- |
| 46 | 46 | 44 | 39 | 27 | 16 |
| 281 | 264 | 246 | 196 | 148 | 90 |
| 0 | 12 | 24 | 36 | 48 | 60 |
| --- | --- | --- | --- | --- | --- |
| 39 | 37 | 32 | 27 | 24 | 11 |
| 16 | 14 | 12 | 10 | 5 | 1 |
| 0 | 12 | 24 | 36 | 48 | 60 |
| --- | --- | --- | --- | --- | --- |
| 33 | 30 | 29 | 23 | 20 | 10 |
| 45 | 42 | 37 | 32 | 29 | 9 |
| 0 | 12 | 24 | 36 | 48 | 60 |
| --- | --- | --- | --- | --- | --- |
| 73 | 68 | 62 | 47 | 40 | 24 |
| 143 | 122 | 103 | 77 | 52 | 28 |
OS
(Survival)
HR: 2.71 [0.25-29.89]
P=0.397
HR: 0.24 [0.1-0.62]
P=0.001
HR: 0.21 [0.03-1.57]
P=0.095
HR, -
P=0.610
| (Months) | |
| --- | --- |
| | |
| (Months) | |
| --- | --- |
| | |
| (Months) | |
| --- | --- |
| | |
| (Months) | |
| --- | --- |
| | |
Number at risk
| Mo |
| --- |
| pCR(+) |
| pCR(-) |
| 0 | 12 | 24 | 36 | 48 | 60 |
| --- | --- | --- | --- | --- | --- |
| 23 | 23 | 23 | 39 | 27 | 16 |
| 290 | 275 | 268 | 196 | 148 | 90 |
| 0 | 12 | 24 | 36 | 48 | 60 |
| --- | --- | --- | --- | --- | --- |
| 26 | 26 | 22 | 19 | 17 | 7 |
| 29 | 25 | 24 | 21 | 14 | 7 |
| 0 | 12 | 24 | 36 | 48 | 60 |
| --- | --- | --- | --- | --- | --- |
| 18 | 17 | 17 | 14 | 12 | 7 |
| 60 | 56 | 50 | 43 | 39 | 14 |
| 0 | 12 | 24 | 36 | 48 | 60 |
| --- | --- | --- | --- | --- | --- |
| 58 | 55 | 51 | 41 | 34 | 22 |
| 155 | 137 | 119 | 90 | 59 | 31 |
